# Bibliographic

**Published:** 1872-12

**Authors:** 


### Bibliographic.

The Pathology of the Teeth; with special reference to their Anatomy and Physiology. By Carl Wedl, M. D., Professor of Histology in the University of Vienna. Translated from the German by W. E. Boardman, M. D.; with notes by T. B. Hitchcock, M. D., D. M. D.; Professor of Dental Pathology and Therapeutics in Harvard University. With one hundred and five illustrations. Published by Lindsay and Blakiston, 1872.

The want of such a work as this has long been felt by the dental profession. There has hitherto been no systematic work upon the pathology of che teeth, coming up to the present attainments upon the subject.

The work consists of two general divisions: first, Anatomy and Physiology; and second, Pathology; apresentation of the former being necessary for the proper elucidation of the latter. There has been no work presented to the profession that so thoronghly occupies the ground as this, which gives special attention to most if not all the diseases and abnormal conditions that attach to the teeth; discussing each fully and clearly; dealing with facts rather than hypotheses.

This work should be in the hands of every dentist, and receive a thorough examination. The work deals with many plain, palpable things, of great importance, that have hitherto been almost wholly ignored.

We advise every one to procure the work, and then study it wholly and thoroughly.

